# Developmental changes in the expression of creatine synthesizing enzymes and creatine transporter in a precocial rodent, the spiny mouse

**DOI:** 10.1186/1471-213X-9-39

**Published:** 2009-07-01

**Authors:** Zoe Ireland, Aaron P Russell, Theo Wallimann, David W Walker, Rod Snow

**Affiliations:** 1 Department of Physiology, Monash University, Clayton, Australia 3800; 2 Centre for Physical Activity and Nutrition Research (C-PAN), School of Exercise and Nutrition Sciences, Deakin University, Burwood, Australia; 3 ETH-Zurich, Institute of Cell Biology, Hoenggerberg, Zurich, Switzerland

## Abstract

**Background:**

Creatine synthesis takes place predominately in the kidney and liver via a two-step process involving AGAT (L-arginine:glycine amidinotransferase) and GAMT (guanidinoacetate methyltransferase). Creatine is taken into cells via the creatine transporter (CrT), where it plays an essential role in energy homeostasis, particularly for tissues with high and fluctuating energy demands. Very little is known of the fetal requirement for creatine and how this may change with advancing pregnancy and into the early neonatal period. Using the spiny mouse as a model of human perinatal development, the purpose of the present study was to comprehensively examine the development of the creatine synthesis and transport systems.

**Results:**

The estimated amount of total creatine in the placenta and brain significantly increased in the second half of pregnancy, coinciding with a significant increase in expression of CrT mRNA. In the fetal brain, mRNA expression of AGAT increased steadily across the second half of pregnancy, although GAMT mRNA expression was relatively low until 34 days gestation (term is 38–39 days). In the fetal kidney and liver, AGAT and GAMT mRNA and protein expression were also relatively low until 34–37 days gestation. Between mid-gestation and term, neither AGAT or GAMT mRNA or protein could be detected in the placenta.

**Conclusion:**

Our results suggest that in the spiny mouse, a species where, like the human, considerable organogenesis occurs before birth, there appears to be a limited capacity for endogenous creatine synthesis until approximately 0.9 of pregnancy. This implies that a maternal source of creatine, transferred across the placenta, may be essential until the creatine synthesis and transport system matures in preparation for birth. If these results also apply to the human, premature birth may increase the risk of creatine deficiency.

## Background

The creatine/phosphocreatine (PCr) system plays an essential role in cellular energy homeostasis, serving as a spatial and temporal energy buffer in cells with high and fluctuating energy demands (for detailed reviews see [1-4]). In adult humans, about half of the creatine requirement is obtained from the diet, with the remainder synthesized endogenously in a two-step sequence involving AGAT (L-arginine:glycine amidinotransferase) and GAMT (guanidinoacetate methyltransferase). The first step involving AGAT occurs mostly in the kidney where arginine and glycine form guanidinoacetate, which later undergoes methylation to form creatine, occurring mostly in the liver via the actions of GAMT.

From the liver, creatine is carried in the blood to creatine-requiring tissues, where it is transported into cells against a large concentration gradient by a creatine-specific, high affinity, sodium- and chloride-dependent creatine transporter protein (CrT) located at the plasma membrane [2,5]. Once inside the cell, creatine kinase regulates the phosphorylation of creatine.

The recently discovered congenital defects in humans affecting creatine synthesis (AGAT or GAMT deficiency), or creatine uptake (CrT deficiency), are characterized by a severe depletion of cerebral creatine/PCr [6]. In early infancy, these patients often show neurodevelopmental delay, mental retardation, inability to speak, epileptic seizures, autism, movement disorders, and are prone to developmental myopathies [7-9]. No amount of creatine supplementation can improve clinical outcomes in CrT deficient patients [7,10]. In AGAT-deficient patients, long-term high dose creatine supplementation offers a clear therapeutic benefit, whereas in GAMT-deficient patients, in order to reduce accumulation of toxic guanidinoacetate, creatine supplementation has to be accompanied by arginine restriction and ornithin supplementation to be effective [11]. Two recent case studies suggest pre-symptomatic creatine supplementation may completely prevent the neurological sequelae when treatment is initiated within 1–4 months of birth, although long term progress is yet to be monitored [12,13].

The reported success of this early intervention creatine supplementation suggests that the fetus only becomes depleted of cerebral creatine after birth. It may be that the mother and/or placental unit sustain the fetal creatine requirement for all of pregnancy [12,13]. The human placenta is known to express CrT RNA [14], and the capacity for maternal-to-fetal transfer of creatine occurs from at least 13 weeks of gestation onwards [15]. In the pregnant rat, such creatine transfer occurs from at least 14 days gestation [15], and the placenta and fetus show an increasing capacity for creatine accumulation (relative to maternal plasma) with advancing gestation [16]. These results suggest that the placental creatine content probably increases with gestation, possibly in conjunction with an increase in the expression or activity of the CrT, however this has not been shown in any species.

Very little is known of the fetal requirement for creatine and how this may change with advancing pregnancy and into the early neonatal period, particularly for tissues known to have a high creatine requirement in the adult (e.g. brain, heart, skeletal muscle) [2]. Braissant and colleagues have shown that in the embryonic rat CrT mRNA is expressed in almost all tissues, including the brain, from as early as embryonic day (E) 12.5 [17]. The brain shows a marked increase in expression of CrT at E15.5 (term is approximately 21 days). These authors did not measure the content of creatine in the developing fetus, so it remains unknown whether and how the pattern of CrT expression actually relates to brain creatine levels.

In the adult mouse brain, CrT expression at the blood brain barrier has been shown to be a major pathway for supplying creatine to the brain [18]. However, neurons, astrocytes and oligodendrocytes in the adult rat brain have been shown to express the creatine synthesizing enzymes, implying that at least some of the brain requirement for creatine is met by *de novo *synthesis [17,19]. In the developing rat brain, AGAT mRNA can be detected in isolated cells of the central nervous system (CNS) from E12.5 onwards, although GAMT mRNA expression is still only barely detectable at E18.5 [17]. It would appear that at the time of birth the rat pup has only a very limited capacity for creatine synthesis within the CNS. It is necessary to understand how these expression patterns change in the postnatal period, as the newborn rat pup does not reach a comparable stage of development to the newborn human infant until at least postnatal day 7 [20,21].

In preparation for birth it is probable that, as for AGAT and GAMT activities in the adult, the fetal kidney and liver must develop an independent capacity for creatine synthesis. In the developing rat, GAMT mRNA expression in the liver shows a steady increase in expression between E12.5–18.5, whereas AGAT mRNA in the kidney is not detectable until E18.5 [17]. These results suggest the altricial rat pup attains the capacity for creatine synthesis only shortly before birth.

Previous studies in rodents have provided insight into the temporal development of the fetal creatine synthesis and transport system [17,22,23]. However, these findings have not been related to the creatine content of fetal tissues and the role of the placenta has not been considered. Due to the relative immaturity of the newborn rat, the changes leading up to birth do not appropriately reflect the changes that are likely to occur in the human during the transition from late gestation to early postnatal life.

The spiny mouse *(Acomys cahirinus) *is a precocial species that can be considered an appropriate animal model for perinatal development in the human. Unlike conventional rats and mice, the spiny mouse has a long gestation (38–40 days), small litter size (1–5, usually 3), and is developmentally more advanced at birth; the body is covered with fur, eyes and ears are functional, they show active olfaction and are capable of thermoregulation and coordinated locomotion [24]. The developmental profiles of the lung [25], liver [26], small intestine and pancreatic enzymes [27], and the completion of nephrogenesis in the kidney before term [28], indicate that, as in the human, organogenesis is largely complete by the end of gestation.

Using the spiny mouse as a model of human perinatal development, the purpose of the present study was to comprehensively examine the development of the creatine synthesis and transport systems. We measured the creatine content of fetal and placental tissues, and sought to determine if the fetus had the capacity to meet its creatine requirement independently of a maternal-placental source.

## Methods

### Animals

All experiments were approved in advance by Monash University School of Biomedical Sciences Animal Ethics Committee, and conducted in accordance with the Australian Code of Practice for the Care and Use of Animals for Scientific Purposes. The spiny mice used in this study were obtained from our own laboratory colony and housed, bred and time-mated as previously described [29].

### Tissue preparation

Placental and fetal tissues were collected at gestational days 20, 25, 30, 34 and 37, and neonatal tissues on postnatal days 2 and 10, from at least 4 different litters for each age (litter size range 2–4). Placentas and fetal and neonatal brain, heart, liver and kidneys were dissected, weighed and snap frozen in liquid nitrogen and stored at -80°C. Heart samples were collected only from gestational day 25 and kidney samples from day 30 due to the limited mass of tissue.

### Tissue creatine

The concentration of creatine and PCr were measured on gestational days 20, 30, 34, 37 and postnatal day 10, as previously described [30]. Briefly, tissues from 4 fetuses/neonates of different litters were weighed (wet mass), freeze dried for 24–48 h, powdered and re-weighed (dry mass). Powdered samples (1–4 mg dry mass) were extracted on ice using 0.5 M perchloric acid and 1 mM ethylenediaminetetraacetic acid, and neutralized with 2.1 M potassium hydrogen carbonate. Samples were assayed for creatine and PCr using enzymatic analysis with fluorometric detection [31]. Due to insufficient tissue mass after freeze drying, measures were not taken for the heart, liver or kidney on the earliest gestational time point at day 20. The estimated amount of total creatine (TCr; creatine + PCr) was determined as: sample tissue TCr concentration × (sample tissue dry mass/sample tissue wet mass) × total tissue wet mass.

### Real-time PCR

Real-time polymerase chain reaction (qPCR) was used to measure mRNA expression of CrT (in placenta, brain and heart), AGAT (in placenta, brain and kidney), and GAMT (in placenta, brain and liver) on gestational days 20, 25, 30, 34, 37, and postnatal days 2 and 10, from 4 animals (of different litters) at each age.

Total RNA was extracted and DNase treated using the commercially available RNeasy Kits (Qiagen, Australia) for all samples except heart, which were extracted using PerfectPure RNA Fibrous Tissue Kit (5 Prime, USA). Sample RNA (0.5–1.0 μg) was reversed transcribed to form cDNA using AMV reverse transcriptase and Random Primers according to the manufacturer's instructions (Promega, USA), and diluted 1:2 with nuclease-free water.

CrT, AGAT, GAMT and 18S primers (see Table 1) were designed based on homologous regions across human, mouse and rat nucleotide sequences (Ensembl Genome Browser) using web based software Primer3Plus [32] and NetPrimer (PREMIER Biosoft International). Optimum annealing temperatures for each set of primers were determined using a primer annealing temperature gradient (range 55.2–65.1°C). All samples were measured in triplicate, and each plate included a calibrator sample and a reaction containing no template (negative control).

**Table 1:** Sequence of forward and reverse primers for genes of interest and housekeeping genes

Gene	Forward Primer Sequence (5'-3')	Reverse Primer Sequence (5'-3')
18S	ACACGGACAGGATTGACAGA	CAAATCGCTCCACCAACTAA
CrT	TCCTGGCACTCATCAACAG	ATGAAGCCCTCCACACCTAC
AGAT	TCACGCTTCTTTGAGTACCG	TCAGTCGTCACGAACTTTCC
GAMT	TGGCACACTCACCAGTTCA	AAGGCATAGTAGCGGCAGTC
β-actin	GACAGGATGCAGAAGGAGATTACT	TGATCCACATCTGCTGGAAGGT
Cyc A	CTGATGGCGAGCCCTTG	TCTGCTGTCTTTGGAACTTTGTC

CrT, creatine transporter; AGAT (L-arginine:glycine amidinotransferase); GAMT, (guanidinoacetate methyltransferase); Cyc A, cyclophilin A.

For all samples except heart, qPCR was performed using an Eppendorf Mastercycler^® ^ep realplex S with RealMasterMix SYBR ROX (5 Prime, USA). Each 20 μl reaction contained 1–3 μl template (1 μl for CrT; 3 μl for AGAT and GAMT) and 0.5 μM of each forward and reverse primer. A 3-step PCR was used to amplify mRNA with an initial template denaturing of 95°C for 2 min, followed by 40 cycles of; 95°C for 15 sec, 64.4, 55.4 or 59.6°C for 15 sec (CrT, AGAT and GAMT, respectively), and 68°C for 20 sec. A fourth step of 80.5°C for 20 sec was included when amplifying AGAT and GAMT mRNA to remove primer-dimer artefact that occurred with low expression of the genes of interest. Heart samples were assayed for CrT using a Stratagene MX3000p thermal cycler system with SYBR Green PCR Mastermix (Applied Biosystems, USA). Each 20 μl reaction contained 2 μl template and 0.2 μM of each forward and reverse primer. A 3-step PCR was used to amplify mRNA; initial template denaturing of 95°C for 10 min, and 40 cycles of; 95°C for 30 sec, 60.0°C for 60 sec, and 72°C for 30 sec. Fluorescence readings were measured during the last step of cycling.

**Figure 1:**
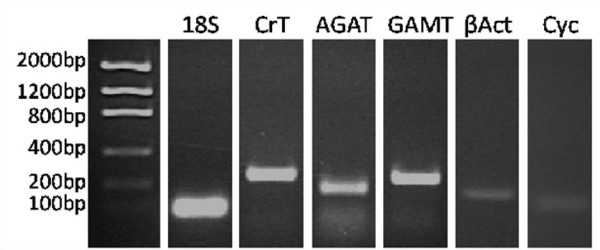
**Specificity of primers for genes of interest and housekeeping genes**. A single DNA product was detected at the expected size for each set of primers; 18S = 86 bp, CrT = 250 bp, AGAT = 182 bp, GAMT = 245 bp, β-actin = 142 bp, cyclophilin A = 67 bp. bp, base pairs; βAct, β-actin; Cyc, cyclophilin A.

A melt curve of fluorescence versus temperature was performed after each qPCR to ensure a single product had been amplified per primer set. The DNA product of each gene of interest, housekeeping gene, and negative controls were run on a 2 percent agarose gel to confirm single product at the expected size (Figure 1).

Data were analyzed and differential expression determined using the comparative ΔΔC_T _(cycle of threshold fluorescence) method. Briefly, relative expression in each sample were calculated by subtracting the mean C_T _value for 18S from the mean C_T _value of the gene of interest; ΔC_T _value. The mean ΔC_T _value of the calibrator sample was then subtracted from each individual sample to give ΔΔC_T_. This number was inserted into the formula 2^-ΔΔCT ^and divided by the mean 2^-ΔΔCT^value of the 37 day gestation group, therefore expressed relative to the mean of the 37 day gestation group for the gene of interest within each organ. The expression stability of the housekeeping gene 18S between gestational day 20 and postnatal day 10 was verified for all organs of interest against β-actin and cyclophilin A using geNorm (internal control gene-stability measure for 18S <1.2 for all organs) [33].

### Immunoblotting

**Figure 2:**
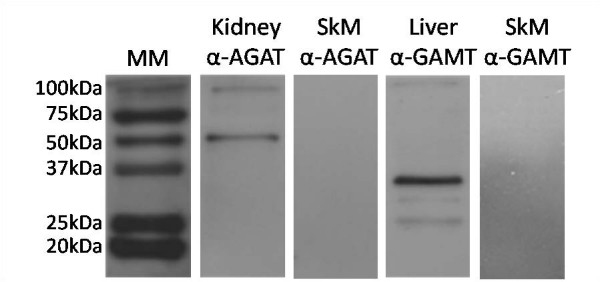
**Specificity of anti-AGAT and anti-GAMT antibodies in spiny mouse tissue**. Antibodies were reactive for a positive band at the expected size, with no positive reaction observed in the negative control sample (skeletal muscle). MM, molecular mass marker; SkM, skeletal muscle.

Western blotting technique was used to measure AGAT protein expression in placenta, brain, and kidney, as well as GAMT protein expression in placenta, brain, and liver homogenates from 4 animals of different litters at select ages between 20 days gestation and postnatal day 10. The proteins were detected with affinity purified rabbit monoclonal (GAMT) and polyclonal antibodies (AGAT) made through injection of the following antigenic peptides: GAMT N-terminal aa 125–145; and AGAT N-terminal aa 62–77 and 410–423. Specific immunoglobulins against AGAT and GAMT were obtained by peptide affinity chromatography. The antibodies detected a positive band at the predicted molecular mass (AGAT, 46 kDa; GAMT, 31 kDa; see Figure 2). No signal was detected in the negative control sample (adult skeletal muscle, [1]).

There has been considerable difficulty in quantifying CrT protein. This is largely due to glycosylation and the difficulties associated with hydrophobic proteins in gels [34], but also because of non-specific immunoreactivity of several anti-CrT antibodies [35]. These antibodies have been shown to cross-react with non-CrT proteins, in particular E2 components of mitochondrial dehydrogenases [35]. We generated a rabbit monoclonal antibody against the CrT N-terminal aa 14–27. In the spiny mouse, the antibody labelled more than one band within the predicted molecular mass range (50–75 kDa, data not shown). As yet we have been unable to verify if one or more of these bands is indeed recognizing the genuine CrT. For these reasons, only mRNA data could be obtained for the CrT.

Protein was extracted using Cell Lysis Buffer (Cell Signalling Technology, USA) and 1 mM serine protease inhibitor phenylmethylsulphonyl fluoride (PMSF). Protein concentrations were determined using the Lowry method with a bovine serum albumin (BSA) standard curve. Samples were prepared with Laemmli sample buffer (0.225 M Tris-HCl pH 6.8, 50% glycerol, 5% SDS, 0.05% bromophenol blue, 0.25 M DTT) and heated at 95°C for 5 min, denaturing the tertiary structure. Prepared samples (10 μg) and 2 μl broad range molecular mass marker were separated using 15% SDS-PAGE gel, and wet electro-transferred (Mini Trans-blot, BioRad Laboratories, Australia) to nitrocellulose blotting membrane. Transfer to membranes was verified with reversible Ponceau S, and gels stained with Coomassie Blue. Membranes were blocked for 120 min in 5% BSA in 0.1% Tween-20 TBS (20 mM tris, 500 mM NaCl, pH 7.4; TBST), and incubated in primary antibody with 5% BSA in TBST overnight at 4°C (anti-AGAT, 1:1000; anti-GAMT, 1:2000). Following incubation, membranes were washed in TBST, incubated with HRP-conjugated goat-anti-rabbit secondary antibody (1:5000, Santa Cruz) and StrepTactin-HRP conjugate (1:10,000) with 5% BSA in TBST at room temperature for 60 min, and given a final wash in TBS. All incubations were performed with gentle agitation. Reactive protein were detected with chemiluminescence (Cell Signalling Technology, Australia) and exposed to X-ray film (Kodak, Australia). Each blot contained a positive control sample, and samples from 2 animals of each age group. Western blot data were analyzed using ImageJ 1.40 g, with values expressed relative to the positive control sample.

### Reagents

Unless otherwise specified, all reagents were obtained from BioRad Laboratories (Australia).

### Statistical analysis

All data are presented as mean ± SE, and were analyzed using a one-way analysis of variance with Tukey HSD post hoc. Significance was set at p < .05. Statistical comparisons were carried out using the computer based program SPSS.

## Results

### Organ growth and creatine

**Figure 3:**
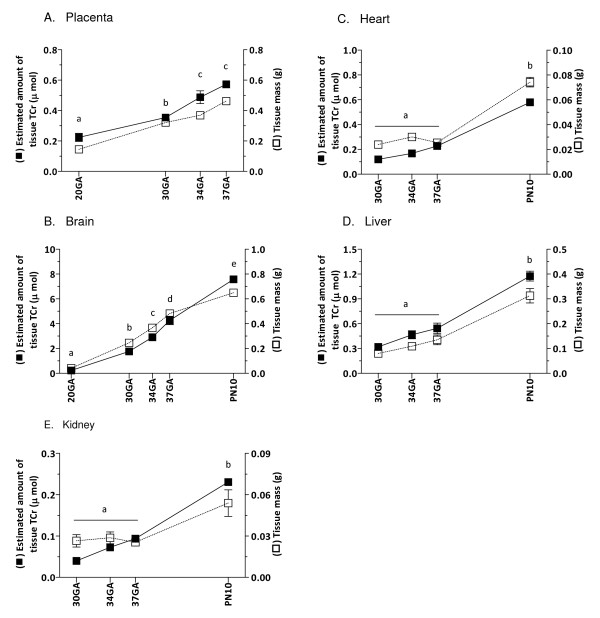
**The estimated amount of tissue TCr (■) and wet mass (□) of placental, fetal and neonatal tissues during development**. A, Placenta; B, Brain; C, Heart; D, Liver; E, Kidney. Data points not sharing the same symbol indicate amount of tissue TCr is significantly different to all others (p < .05). Mean ± SE. TCr, total creatine; GA, gestational days; PN, postnatal days.

Figure 3A–E shows the mass and estimated amount of tissue TCr in the placenta from mid-gestation until birth, as well as in brain, heart, liver and kidney from mid-gestation to postnatal day 10. The estimated amount of TCr in the placenta and brain showed a steady and significant increase between 20–37 days gestation (p < .05, Figure 3A–B). In the brain, the TCr content continued to increase in the postnatal period. Although the tissue mass increased with age as expected for all organs, the estimated amount of tissue TCr did not change in the fetal heart, liver and kidney between 30–37 days gestation, but it had increased significantly by postnatal day 10 (p < .05, Figure 3C–E).

### Enzymes of creatine synthesis in the kidney and liver

**Figure 4:**
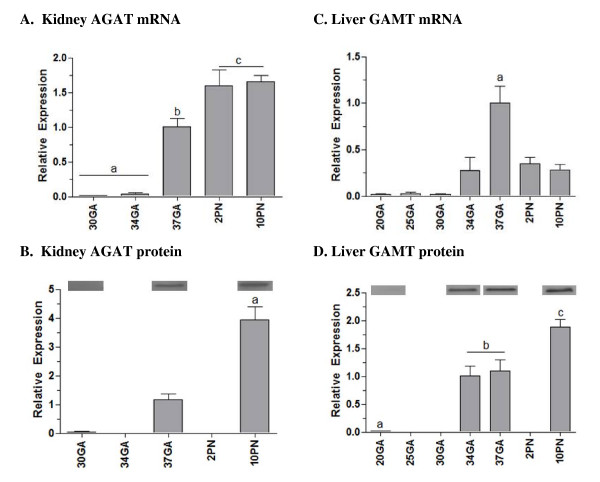
**Expression of the creatine synthesizing enzymes AGAT and GAMT in the kidney and liver during fetal and neonatal development**. **A**, Kidney AGAT mRNA expression; **B**, Kidney AGAT protein expression; **C**, Liver GAMT mRNA expression; **D**, Liver GAMT protein expression. All mRNA data are normalized to 18S and shown relative to 37 days gestation. Data points not sharing the same symbol indicate expression is significantly different to all others (p < .05). Mean ± SE. GA, gestational days; PN, postnatal days.

The developmental expression of the two key enzymes involved in creatine synthesis, AGAT and GAMT, were measured in the kidney and liver, respectively, at the mRNA and protein level from mid-gestation until the second postnatal week (Figure 4A–D). Expression of AGAT mRNA in the kidney remained relatively low between 30 and 34 days of gestation, with a significant 33-fold increase in expression by 37 days of gestation (p < .05, Figure 4A). A further increase had occurred by postnatal day 2 (p < .05), with no further change by postnatal day 10. The expression of AGAT protein showed a similar profile; protein levels significantly increased between gestational days 30, 37 and postnatal day 10 (p < .05, Figure 4B).

There was relatively low expression of hepatic GAMT mRNA between gestational days 20–30, with a significant 50-fold increase in expression by day 37 of gestation (p < .05, Figure 4C). Expression was lower after birth, with the postnatal mRNA levels being similar to that at 34 days of gestation. The expression of GAMT protein increased significantly between gestational days 20 and 34–37, similar to the mRNA profile, however a further significant increase in GAMT protein occurred by postnatal day 10 (p < .05, Figure 4D).

### Enzymes of creatine synthesis in the brain

**Figure 5:**
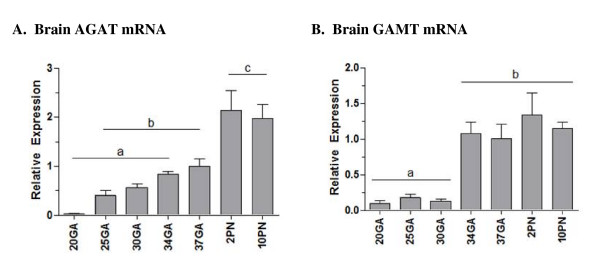
**Expression of the creatine synthesizing enzymes AGAT and GAMT in the brain during fetal and neonatal development**. **A**, Brain AGAT mRNA expression; **B**. Brain GAMT mRNA expression. All mRNA data are normalized to 18S and shown relative to 37 days gestation. AGAT and GAMT protein could not be detected with western blot, possibly due to low level expression in whole brain homogenates. Data points not sharing the same symbol indicate expression is significantly different to all others (p < .05). Mean ± SE. GA, gestational days; PN, postnatal days.

AGAT mRNA expression showed a gradual increase between gestational days 20, 25, 30, 34 and 37, although only reached significance between days 20 and 37 (p < .05, Figure 5A). A further 2-fold increase in AGAT mRNA expression occurred by postnatal day 2 (p < .05), and remained unchanged at postnatal day 10. Expression of GAMT mRNA in the fetal brain was relatively low between 20–30 days gestation, with a significant 10-fold increase by 34 days gestation (p < .05, Figure 5B). Levels remained unchanged by 37 days gestation and into the postnatal period. Although mRNA for both AGAT and GAMT were detected, the corresponding proteins could not be detected by Western blot analysis (data not shown).

### Enzymes of creatine synthesis in the placenta

AGAT and GAMT could not be detected in any placenta samples between 20 and 37 days of gestation at the mRNA or protein level (data not shown).

### Creatine transporter

**Figure 6:**
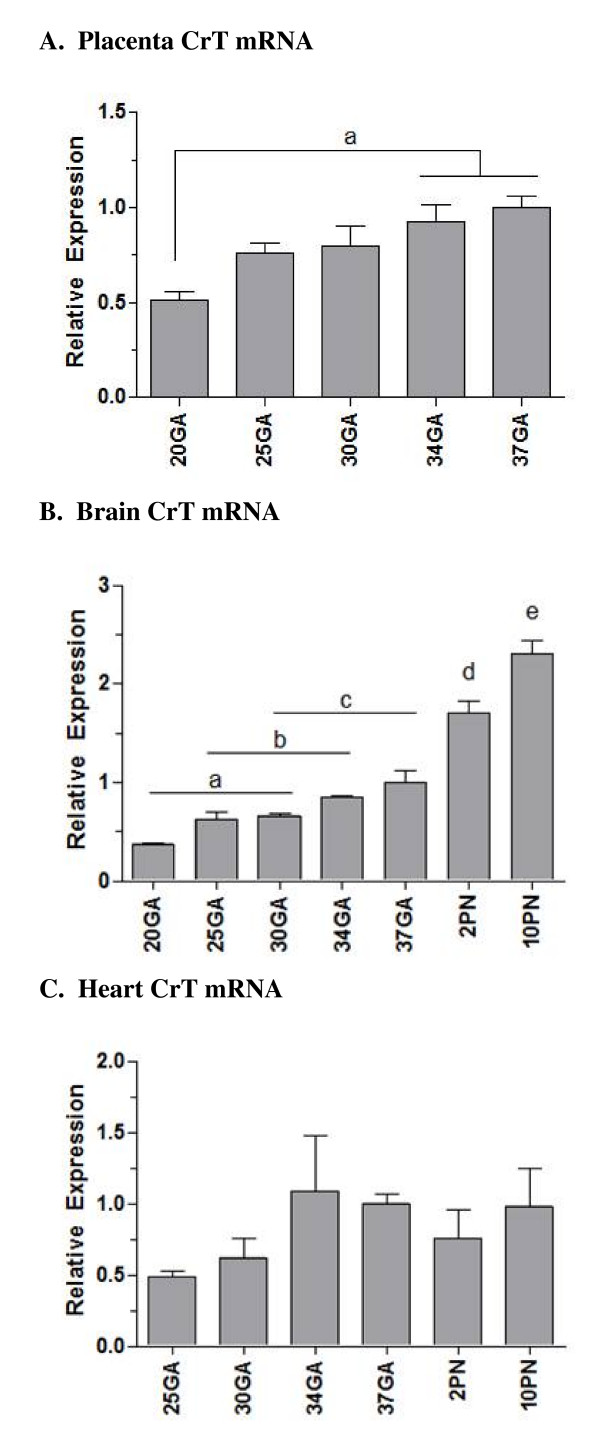
**Creatine transporter mRNA expression in the developing spiny mouse placenta, brain and heart**. **A**, Placental CrT mRNA expression; **B**, Brain CrT mRNA expression; **C**, Heart CrT mRNA expression. All mRNA data are normalized to 18S and shown relative to 37 days gestation. Data points not sharing the same symbol indicate expression is significantly different to all others (p < .05). Mean ± SE. GA, gestational days; PN, postnatal days.

The expression of CrT mRNA was determined from mid-gestation until postnatal day 10 (Figure 6). In the placenta and brain, CrT mRNA expression was detected early on in pregnancy and showed a significant 2-fold increase in expression from gestational day 20 to 37 (p < .05, Figure 6A–B). In the brain, a further increase occurred postnatally, with CrT mRNA increasing a further 2.3-fold between late gestation (day 37) and postnatal day 10. In the heart, CrT mRNA increased approximately 2-fold between gestational days 30–34, although this did not reach significance (P = 0.35; Figure 6C).

## Discussion

The provision of creatine to the tissues of a developing embryo is likely to be important for normal fetal development, particularly for energy homeostasis in the brain and heart. Despite this, very little is known about when creatine synthesis and its transporter system develops during embryonic, fetal and neonatal life. In this study we used a species that, like the human, has a relatively long gestation during which considerable fetal development and maturation occurs. We determined that the amount of TCr in the spiny mouse placenta and fetal brain increased progressively across the second half of gestation. However, expression of the two principal creatine synthesizing enzymes AGAT and GAMT in the fetal kidney, liver and brain was low until very late in gestation (days 34–37), and expression of these enzymes in the placenta was not detected at all. These results suggest that in precocial species the developing fetus is almost completely reliant on a maternal source of creatine until as late as 0.9 of pregnancy.

The fetal brain showed a steady and significant increase in the estimated amount of TCr from mid-gestation until term, and increased further in the postnatal period. The high amount of creatine found in this organ is not surprising, as the adult brain is known to have a high basal creatine concentration, presumably to cope with its large and fluctuating cellular energy requirements [3,4].

In the adult rat brain, there is a limited capacity for creatine to cross the blood brain barrier. The widespread expression of creatine synthesizing enzymes has lead to the suggestion that the creatine requirements of the brain can be met, at least in part, independently of extra-CNS sources (i.e. that synthesized via the kidney and liver) [19]. In the spiny mouse, although AGAT and GAMT mRNA could be measured in whole brain extracts, protein expression could not be detected with western blot analysis. On the basis of this result, it could be argued that the fetal and neonatal brain does not have the capacity to synthesize creatine in significant amounts on a whole organ level. A more likely explanation is that, as for the rat, AGAT and GAMT protein expression in the fetal spiny mouse brain is region and cell-specific [17]. Immunoblot with whole brain homogenate is most likely too insensitive to detect such protein expression.

The temporal difference in the appearance of AGAT and GAMT mRNA in the fetal spiny mouse brain is an interesting phenomenon. A similar result was found for the embryonic rat, where AGAT could be detected from E12.5, yet GAMT was barely detectable even shortly before birth at E18.5 [17]. These results suggest that the fetal brain relies on extra-CNS or maternal sources of creatine for the whole of pregnancy, and/or the creatine precursor guanidinoacetate is transferred from the CNS to GAMT-expressing cells where it can be converted to creatine. Although our results have not been confirmed at the protein level, the expression patterns of AGAT and GAMT mRNA suggest that the fetal spiny mouse brain does not attain an appreciable capacity for significant creatine synthesis until shortly before birth, at 34–37 days gestation (0.9 of pregnancy).

We showed that CrT mRNA expression in the spiny mouse brain increased approximately 2-fold between mid-pregnancy and term; we were unable to measure CrT protein for lack of an appropriately specific antibody (as detailed in Methods). This prenatal increase is in agreement with that described in the embryonic rat [17], and consistent with its early expression in zebra fish [36]. Further increases in CrT expression in the spiny mouse brain were observed at 2 and 10 days after birth. In the neonatal rat brain, the concentration of creatine and creatine kinase has been reported to increase significantly between postnatal weeks 1 and 3, with levels essentially remaining unchanged after that [37,38]. It is likely that the pre- and postnatal developmental increase in brain CrT expression in the spiny mouse coincides with the increasing demand for creatine in the maturing CNS, which cannot be met entirely by creatine synthesis within the CNS.

AGAT and GAMT expression in the fetal kidney and liver were also relatively low until the very late stages of pregnancy; a 30 to 50-fold increase in mRNA expression was seen at 34–37 days of gestation (term is ~39 days). We expected that in preparation for birth these key organs would develop a capacity for creatine synthesis in the latter half of pregnancy, but these results suggest a limited capacity for endogenous creatine synthesis exists until very late (~0.9) in pregnancy. AGAT and GAMT expression levels in the kidney and liver remained unchanged between gestational day 37 and postnatal day 10. It would appear that shortly before birth, the spiny mouse attains the capacity to meet the postnatal requirement for endogenous creatine synthesis. It would be interesting to know how the uptake of creatine from breast milk contributes to the neonatal requirement for creatine in the spiny mouse.

Similar to the brain, the amount of TCr measured in the placenta of the spiny mouse increased significantly with advancing pregnancy. Being metabolically very active, it is likely that the placenta itself has a requirement for creatine – creatine kinase expression, which is tightly coupled with cellular energy requirements, peaks in term human placenta [39]. However, as for the human placenta [4], it appears that this organ itself does not synthesize creatine, as we were unable to detect either AGAT or GAMT mRNA or protein in the placenta of the spiny mouse from mid-gestation to term. The increase in placental TCr may reflect an increase in a temporary pool of 'stored creatine' available for transfer to the fetus, which is consistent with our observation that there was an increase of CrT mRNA in the placenta from at least mid-gestation. At the present time we do not know whether this occurs in maternal or fetal tissue in the placenta. The increase coincides with the development of the labyrinth region of the placenta, which is primarily fetal tissue and associated with the rapid expansion of the fetal vascular compartment [40]. An increase in placental CrT may allow for more efficient transfer of creatine into the fetal circulation with increasing gestation – thus meeting the fetus' growing demand for creatine, particularly that of the fetal brain.

To our knowledge, the transcriptional pathways controlling the regulation of the CrT, AGAT and GAMT genes have not been identified. In the fetal spiny mouse, circulating thyroid hormone increases steadily between 30 days gestation and term [41]. Analysis of the CrT promoter reveals approximately six nuclear respiratory factor 1 (NRF1) consensus sequences, for which thyroid hormone is a known activator of [42]. It is plausible that the CrT gene is regulated, at least in part, via a thyroid hormone/NRF1 transcriptional program, however this is yet to be established.

Although the fetal heart, liver and kidney undergo considerable growth from mid-gestation until term in the fetal spiny mouse, the estimated amount of TCr did not increase until after birth. Although heart muscle does not synthesize creatine, as with skeletal muscle it has a large requirement for creatine and therefore a considerable capacity for uptake and storage [1]. Our finding that heart TCr did not increase until the postnatal period is in agreement with previous studies in the rat, where creatine levels, as well as creatine kinase levels, increased 5-fold in the 3 weeks after birth [37,38]. As cardiovascular function is of fundamental importance for growth from very early in pregnancy [43], it is not altogether surprising that cardiac creatine levels are relatively high and stable throughout gestation. The quantity of creatine found in the heart from gestational day 30 is obviously sufficient to sustain cardiac function until term. However, at birth the heart undergoes rapid growth and re-modelling associated with transformation of the circulation with the onset of pulmonary ventilation and closure of the major vascular shunts, the ductus arteriosus and foramen ovale. Thus, between gestational day 37 and postnatal day 10 the mass of the heart increased almost 3-fold, TCr content increased 2-fold, and CrT mRNA expression increased 2-fold between mid-gestation and postnatal day 10 (although this did not reach significance). It is possible that creatine uptake into the heart is facilitated by an increase in CrT activity rather than CrT protein expression, and further increases in transporter expression may occur later than postnatal day 10. In support of this, is the fact that CrT activity can be regulated by phosphorylation via protein kinases [44,45].

Unlike the brain and skeletal muscle, the kidney and liver have less requirement for creatine [46]. The observed rapid increase in TCr in these organs in the postnatal period is likely to reflect their functional maturation. The kidney plays a key role in the re-absorption of creatine from urine, the capacity for which has been shown to increase after birth in both the human and rat [47]. Likewise, the methylation of guanidinoacetate to creatine, a process occurring predominantly in the liver, also appears to increase after birth.

## Conclusion

These results suggest that, for a species where considerable maturation of the fetus occurs before birth, there appears to be a limited capacity for endogenous creatine synthesis until approximately 0.9 of pregnancy. This implies that a maternal source of creatine, transferred to the fetus via the placental unit, may be essential until the creatine/PCr synthesis and transport system matures in preparation for birth. If these results also apply to the human, it may be that infants born prematurely would be at risk of becoming creatine deficient, unless sufficient creatine can be absorbed by the immature gut from the limited amounts of creatine present in breast milk [12]. Organs with the highest energy requirements, such as the brain and heart, would be at greatest risk at this time. Also, fetal growth-retardation arising from chronic placental insufficiency may also result in high risk for creatine deficiency, as our results suggest that the placenta has an essential role in the transfer of creatine from mother to fetus. We have previously shown that creatine supplementation of the maternal diet is of benefit to the fetus when exposed to severe hypoxia at birth [30]. Creatine supplementation may also benefit the growth-retarded fetus, especially since many of these are also born preterm.

## Authors' contributions

ZI carried out all animal studies, molecular work, participated in design of study and drafted the manuscript. APR participated in molecular work and drafting of manuscript. TW designed and synthesized antibodies, provided technical assistance and advice in molecular techniques, and drafting of manuscript. DWW participated in design and coordination of study, and drafted manuscript. RS participated in design and coordination of study, technical assistance, and drafting of manuscript. All authors have read and approved the final manuscript.
